# LINAC‐based stereotactic radiosurgery for treatment of trigeminal neuralgia

**DOI:** 10.1120/jacmp.v5i3.1997

**Published:** 2004-10-21

**Authors:** Bruce J. Gerbi, Patrick D. Higgins, Kwan H. Cho, Walter A. Hall

**Affiliations:** ^1^ Department of Therapeutic Radiology‐Radiation Oncology University of Minnesota Mayo Mail Code 494, 420 Delaware St. S.E. Minneapolis Minnesota 55455 U.S.A.; ^2^ National Cancer Center Center for Proton Therapy Madu 1‐dong Ilsan Goyang Gye onggi 411‐764 Korea; ^3^ Department of Neurosurgery University of Minnesota Mayo Mail Code 96, 420 Delaware St. S.E. Minneapolis Minnesota 55455 U.S.A.

**Keywords:** trigeminal neuralgia, stereotactic radiosurgery, Gamma Knife, linear accelerator, floor stand, facial pain

## Abstract

Trigeminal neuralgia (TN) is a disabling pain condition that has classically been treated using either surgical or medical techniques. Several researchers have shown that stereotactically delivered radiation can be an effective tool in the amelioration of this condition. For these studies, the Gamma Knife was used to deliver the radiation treatment. The target location was designated as the proximal nerve at the root entry zone, and doses greater than 70 Gy to the maximum point in a single fraction were found to be effective in controlling pain in 80% of the patients treated. LINAC‐based stereotactic radiosurgery has been notably absent from the treatment of TN, even though it has many similarities to Gamma Knife‐based stereotactic radiosurgery.

The aim of this paper is to describe our LINAC‐based stereotactic technique for treatment of TN. We also compare treatment of TN using our technique to that using the Gamma Knife. We found that a LINAC‐based treatment of TN can be accomplished with accuracy comparable to treatments delivered using the Gamma Knife. The dose distributions are essentially equivalent for the two treatment approaches. The LINAC‐based system is easy to plan and offers the ability to reduce the involvement of sensitive structures from the treatment fields as well as the Gamma Knife system does. A disadvantage of the LINAC‐based system is the time involved for treatment.

PACS number: 87.53.Tf 87.56.Da

## I. INTRODUCTION

Trigeminal neuralgia (TN) is a disabling pain condition that has classically been treated using either surgical or medical techniques. Recently, research in several publications has shown that stereotactic radiosurgery can be an effective tool in the amelioration of this condition.^(^ ^1^ ^–^ ^8^ ^)^ In the majority of the studies, the Gamma Knife was used to deliver the radiation treatment. The 4‐mm aperture was used for these treatments, and the target location was designated as the proximal nerve at the root entry zone. Doses greater than 70 Gy to the maximum point (100%) in a single fraction were found to be effective in controlling pain in 80% of the patients treated. Patients with no prior surgery had complete or near complete pain relief, while complications due to treatment were nearly nonexistent in the published studies. What makes this particular treatment unique from other stereotactic treatments is the extreme accuracy that is required for all steps for successful treatment. This is primarily due to the small size of the target, the difficulty in identifying the target region, the small cone sizes used for treatment, and the extremely high single‐shot doses >70 Gy that could result in neural injury if they were misguided (or misdirected).

The use of LINAC‐based stereotactic radiosurgery for the treatment of TN has been limited in the historical literature,^(^ ^6^ ^–^ ^8^ ^)^ even though many of the steps in the process of this stereotactic treatment are similar, if not identical, to those undertaken for a Gamma Knife treatment. Due to the effectiveness of this treatment for pain relief, we initiated a program of LINAC‐based stereotactic radiosurgery at our institution. We evaluated our technique to determine if our LINAC‐based system had the required accuracy to properly treat such small target volumes. Positioning accuracy and dose distributions are compared against what is expected for Gamma Knife treatments, and application of LINAC‐based stereotactic radiosurgery for this clinical problem is discussed.

## II. METHODS

We compared the mechanical accuracy of the two systems, the imaging accuracy of the techniques, and the dosimetric aspects of the treatment including coverage of the target, and the dose to uninvolved normal structures.

### A. Description of the two techniques

Our LINAC‐based system uses the Radionics, Inc. MRI‐compatible head ring for patient fixation; the larger diameter Brown‐Roberts‐Wells (BRW) head ring is attached to the MRI head ring for CT scanning and to secure the patient to the treatment stand. Depth helmet measurements are taken after the head ring is fixed initially to the patient and immediately before treatment to ensure that ring placement has remained constant throughout the process. The high‐precision beam delivery is provided by a Philips (Elekta) SRS 200 independent support stand and subgantry assembly installed on a 6‐MV X‐ray Varian 6/100 LINAC. A 5‐mm circular cone is used for the treatments, with the LINAC collimators set for a $5\times 5\;{\text{cm}}^{2}$ field.

The Philips SRS 200 mechanical system, which is the key source of the mechanical accuracy required by this technique, consists of an independent subgantry assembly, which has a head support system that is independent of the treatment couch, and a secondary gantry, which holds the cones for patient treatment. This independent subgantry is mated to the LINAC through a gimbal assembly. The force to rotate the subgantry assembly is supplied by the LINAC and transmitted to the subgantry via the gimbal assembly. Any small misalignment between the LINAC rotation and the subgantry rotation is handled by the play available in the gimbal assembly. This integrated subgantry is the source of the extreme accuracy required for this particular radiosurgery technique. The independent head support is much more accurate for head positioning than the LINAC couch, while the subgantry is much more accurate than the gantry rotation of a standard LINAC. The capabilities of the system are well documented^(9)^ and easily meet the manufacturer's mechanical accuracy design specifications of 0.3 mm average with no more than 0.5 mm deviation at any one point using the tests described in the literature.^(^ ^10^ ^,^ ^11^ ^)^ It is the extreme accuracy of this system that allows for the treatment of TN using a LINAC; couch‐based stereotactic systems are completely dependent on the rotational accuracy of the couch and gantry of the LINAC itself. This Philips SRS 200 system, no longer marketed, is the precursor to the LINAC Scalpel™ System currently available from Zmed, Inc. (recently acquired by Varian Medical Systems, Inc.).

In our procedure, we first obtain MRI scans of the head with the MR head ring and universal localizer (UCLF) on the patient using a 3D data acquisition sequence (MP‐RAGE) on a Siemens Magneton scanner. Author Sagittal, axial, and coronal scans of 1 mm thickness at 1 mm spacing are reconstructed from this data acquisition sequence. CT scans are then acquired with the BRW head ring attached to the MR head ring with the CT fiducial localizer in place. CT scans are obtained at 1 mm spacing through the target region with a Siemens Somatom Plus scanner with 2‐mm scans taken outside the treatment region. Additional checks are performed to ensure the accurate transfer of the MR and CT scan data from the scanners to the treatment‐planning system.

**Figure 1:**
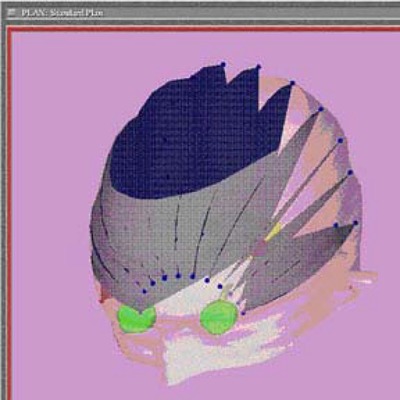
LINAC‐based stereotactic radiosurgery arcs for treating trigeminal neuralgia. Thirteen 6‐MV X‐ray arcs with a total of approximately 1300 arcs degrees of rotation are represented in this plan. The 5‐mm cone is used for treatment, and the arc separation is 15°.

The target is determined using the sagittal and axial MR scans. The target volumes are drawn using the MR image sets obtained with the Radionics universal localizer (UCLF) in place during the MR scans and using the Siemens MR head coil. The MR image sets are transformed into the BRW coordinate system using the fiducials on the MR images. The CT data set is required for planning by the Radionics software, and we use the CT image set to delineate the external contour, the eyes, optic nerves, and brain stem. The treatment field arrangement for our LINAC‐based treatment contains 13 arcs spaced at 15° intervals for approximately 1300 total degrees of arc rotation (Fig. 1). Treatment planning is done to deliver 7000 cGy to 8750 cGy to the maximum using Xknife‐4 software supplied by Radionics™. When the patient is ready for treatment and attached to the independent support stand, we ensure that the plan specified treatment coordinates are properly set.

**Figure 2:**
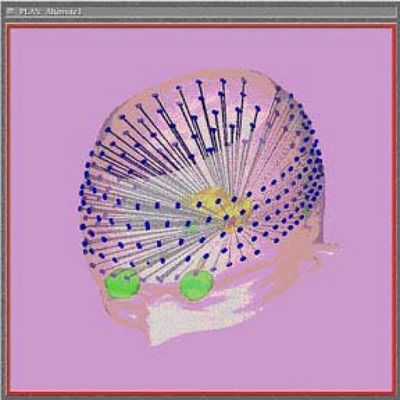
A Gamma Knife B unit simulated using the Radionics™ Xknife software. The 201 4‐mm cobalt‐60 beams are simulated using actual off‐axis ratio, TMR, and beam orientation information for this unit.

To evaluate the dosimetric component of our treatment technique, we compared our planning results to a Gamma Knife B unit that we simulated in our Xknife planning computer. The Gamma Knife unit consisted of 201 individual cobalt‐60 sources arranged radially in five rings of 35, 39, 39, 44, and 44 sources (Fig. 2). We obtained the collimator location and patterns for an installed Gamma Knife B unit along with tissue‐maximum ratio (TMR) and dose profile data for their 4‐mm collimators. This information was entered into the Xknife 4 planning system in the same manner as our LINAC data. Once this Gamma Knife beam data was in the program, it could be used directly in the Xknife planning software in the same manner as the LINAC information. Thus a direct comparison of the Gamma Knife system and our LINAC‐based system could be conducted on the same patient‐related image data sets.

The accuracy of the beam modeling for the Gamma Knife data in Xknife was verified before doing the test comparisons of dose distribution for the LINAC and Gamma Knife trigeminal treatments. We first tested the accuracy of the representation of a single 4‐mm Gamma Knife Co‐60 beam in the Xknife planning system. This Gamma Knife beam was simulated in the Xknife software as a single beam having a one‐degree arc. At the isocenter, the profile obtained from this test matched the data of the input dose profile. The treatment plan of the trigeminal nerve using the Gamma Knife data was simulated in Xknife using 201 single arc beams with the same isocenter location (Fig. 2) as our LINAC‐based plan using 13 arcs. We did not compare the plan using the Xknife representation of a Gamma Knife unit directly to a plan calculated from a Gamma Knife treatment planning computer.

Our treatment technique using 13 arcs totaling approximately 1300 arc degrees of rotation was designed to deliver approximately the same amount of dose along the arc path as that given along a Gamma Knife beam. Our rationale for choosing this beam arrangement was based on the assessment that each of the individual 201 fields of the Gamma knife could be closely approximated by a 5° arc, and that our 1300 degrees of rotation would equal about 260 five‐degree arc segments. Each Gamma Knife cobalt field would essentially deliver 7000 cGy/201 beams, or approximately 35 cGy to the isocenter per beam. 7000 cGy delivered using 260 five‐degree segments would deliver about 27 cGy per 5° arc, close to the 35 cGy per beam for the Gamma Knife unit. Thus the dose through normal brain tissue for each of these 5° segments would be close to the dose delivered along the track of each Gamma Knife beam.

### B. Mechanical accuracy of treatment delivery

#### B.1 Isocenter alignment

The mechanical isocenter accuracy of the SRS 200 system comes from the design, which minimizes the couch inaccuracy effect by supporting the patient's head independent from the LINAC treatment table. In addition, the subgantry system minimizes the inaccuracies of gantry rotation.^(9)^ Using the standard Lutz alignment test,^(10)^ this system exhibits a mechanical accuracy of <0.3 mm deviation average with a maximum deviation of 0.5 mm.

The mechanical isocenter accuracy of the Gamma Knife system is approximately 0.25 mm, as reported in the literature.^(12)^

#### B.2 Frame system accuracy

The application accuracy^(13)^ of the various head frames has been reported in the literature.^(^ ^13^ ^,^ ^14^ ^)^ This represents the total clinically relevant uncertainty and includes image reconstruction accuracy, coordinate system transformation, and mechanical errors. The application accuracy of the Leksell frame used with the Gamma Knife was reported to be 1.7 mm ± 1.0 mm (SD), while the BRW frame was 1.9 mm ± 1.0 mm (SD).^(13)^ The general conclusion of these investigations is that there is no major difference in either accuracy or precision between the Leksell or the BRW frame systems when the CT slice thickness is 1 mm.

#### B.3 Imaging accuracy

**Figure 3:**
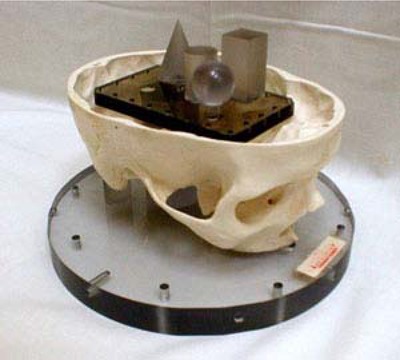
The Radionics™ test phantom showing the sphere, cube, cylinder (in center), and cone (to the back of the image).

Imaging accuracy depends on the scanning system employed, the coordinate transformation accuracy of the planning system, and the accuracy of the transfer of imaging data from the scanner to the planning computer. Imaging accuracy for our system was determined using the Radionics™ head test phantom as the standard of comparison. The Radionics™ test phantom consists of four internal structures—a cube, cone, cylinder, and sphere—whose location in BRW space is very accurately known (Fig. 3). For tests of both the CT and MR scan set accuracy, the phantom was scanned on the appropriate unit, and these scans were transferred to the planning computer. The internal structures of the phantom were contoured, and the location of the tops of these structures in stereotactic space was determined. These identified locations were then compared against the known coordinates of these structures supplied by the manufacturer. The assessment of accuracy is determined as the difference between the expected location of the tops of these structures and the reconstructed *x, y,* and *z* (anterior, lateral, vertical) coordinates of these structures in the transformed computer stereotactic coordinate system.

CT resolution for the Gamma Knife system has been reported to be approximately 1.7 mm.^(12)^ Our tests of CT accuracy done for the BRW system using the Radionics™ test phantom showed a mean difference of 1.1 mm between the expected target coordinates of the test objects and the measured coordinates on images transferred to the Xknife treatment planning computer.

Table 1 shows test results of MR accuracy done using 1‐mm reconstructions from a 3D volume data acquisition sequence (MP‐RAGE) for two Siemens Magneton MR scanners. For this test, the standard Siemens head coil was used with the Radionics™ test phantom secured in the MR head ring and with the UCLF fiducial localizer in place. We found average uncertainties in position of the four phantom test objects ranging from 0.5 mm to 1.2 mm, which is similar to the results for CT reconstruction. The root‐mean‐square error (RMS) for the individual scan orientations (either a coronal, sagittal, or axial scan set) ranged from 1.0 mm to 1.4 mm. Although inaccuracies experienced with stereotactic MR scans are well documented in the literature,^(^ ^15^ ^,^ ^16^ ^)^ our tests of the MR accuracy of the UCLF localizer have consistently yielded the indicated results. For each patient treatment, the reconstructed locations of the localizer frame are checked, and they routinely match the theoretical location of the localizer rods.

**Table 1:** The average difference and the standard deviation (SD) between the expected AP, lateral, and vertical BRW coordinate of the four test phantom objects for MR scans done using the Siemens Magneton MR MP‐RAGE acquisition sequence. These data show the accuracy of the image data transferred and analyzed on the Xknife treatment‐planning computer.

Scan type	AP difference (avg. ± 1 SD)	Lateral difference (avg. ± 1 SD)	Vertical difference (avg. ± 1 SD)	Root‐mean‐square error
MR axial	0.29±0.29 mm	0.31±0.27 mm	0.93±0.44 mm	1.02±0.59 mm
MR coronal	0.32±0.18 mm	0.20±0.19 mm	1.23±0.50 mm	1.29±0.56 mm
MR sagittal	0.33±0.20 mm	0.48±0.36 mm	1.22±0.61 mm	1.35±0.74 mm

## C. Dosimetric comparisons

**Figure 4:**
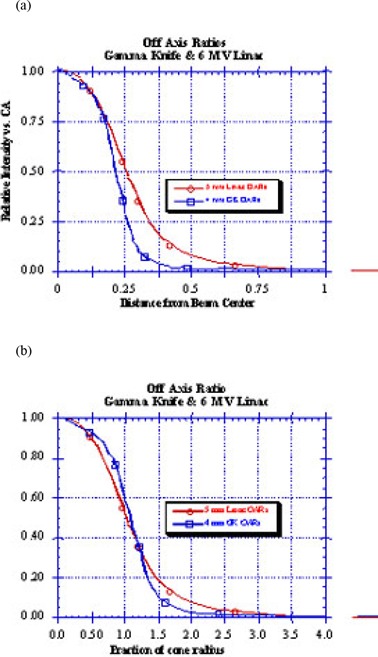
A comparison of the off‐axis ratios for a single 4‐mm Gamma Knife beam versus the 6‐MV X‐ray 5‐mm collimator LINAC beam. (a) The off‐axis ratios plotted as a function of distance from the center of the collimator; (b) the off‐axis ratios normalized to the fraction of the cone radius.

Calculated dose comparisons were done between the 5‐mm, 6‐MV X‐ray LINAC‐based beam and the 4‐mm Co‐60 fields from the Gamma Knife unit. (Figure 4a) shows the off‐axis ratio (OAR) data for the two individual beams as a function of the distance from the center of the field. (Figure 4b) is the same data normalized to the 50% beam intensity location. The penumbral widths (80% ‐ 20%) were 1 mm for the Co‐60 beams and 2.1 mm for the 6‐MV field, indicating that the Gamma Knife beam had a sharper falloff.

A test case on which to make the comparison was a patient treated for TN using the LINAC‐based system. Dose distributions were done for the 13‐arc LINAC‐based treatment approach and compared to the Gamma Knife plan. The locations of the 201 Co‐60 sources were from a B model unit, and the beams were represented as 1° arcs in the treatment‐planning system. As stated above, actual OAR and TMR data from an existing clinical Gamma Knife B unit were input into the system to provide a realistic comparison of delivered dose.

## III. RESULTS AND DISCUSSION

Table 2 shows the calculated dose to the various structures for the LINAC‐based system and the Gamma Knife system when 70 Gy is delivered to the maximum point for each treatment system. The doses to uninvolved structures are generally higher for the LINAC‐based system but not significantly so. From an imaging, mechanical accuracy, dosimetric, and clinical standpoint, we are encouraged by the results of the comparison between our LINAC‐based stereotactic dose delivery system for treatment of TN. Radiobiological concerns are a factor with our LINAC‐based system, since treatment time is typically 2.5 h on a Varian 6/100 running at 200 monitor units (MU) per minute and limited to 2.8 MU/degree. On a more modern LINAC running at 400 MU/min and capable of delivering 10 MU/degree, the treatment time could be reduced by a factor of 3, which would give treatment times more in line with those of a Gamma Knife unit.

**Table 2:** The dose delivered to various structures for the Gamma Knife and LINAC‐based system when 7000 cGy is delivered to the maximum point

	Gamma Knife Dose summary Gamma Knife B unit, Co‐60 gamma rays, 4‐mm collimators, 201 individual sources		LINAC Dose summary LINAC‐based treatment regimen: 6‐MV X‐rays, 5‐mm cone, 13 arcs, ~1300 total arc degrees, 15° spacing between arcs	
anatomical structure	Avg. (cGy)	Min/Max (cGy)	Avg. (cGy)	Min/Max (cGy)
brain stem, axial MR defined	92	13/2746	119	10/3144
optic chiasm, coronal MR defined	58	24/84	76	38/114
left optic nerve, CT defined	16	12/37	10	10/10
right optic nerve, CT defined	14	12/28	10	9/13
left eye, CT defined	13	10/27	9	10/10
right eye, CT defined	13	9/25	9	8/9

## IV. CONCLUSION

By comparison of imaging accuracy, mechanical accuracy, dosimetric comparisons, and clinical results, we feel that LINAC‐based stereotactic radiosurgery using this subgantry assembly is an effective means of treating TN and can be comparable to Gamma Knife treatments.
